# Spasmodic Asthma

**Published:** 1853-09

**Authors:** 


*Spasmodic Asthma.*

A correspondent asks us for the best treatment for spasmodic asthma. The benefit derived from anodynes, antispasmodics and anæsthetics, such as chloroform, ether, &c., is but slight, and at most temporary. Dr. Watson, in the Glasgow Medical Journal, expresses the opinion that in a certain number of cases the affection is dependent on laryngeal disease. By overcoming the contraction at the glottis, that of the small bronchæ either simultaneously or speedily relaxes. The indication in these cases may be fulfilled more or less perfectly by the application to the larynx of a solution of nit. silver of the strength of from xv. to xxx. gs. to the ℥j. of water.

Electricity, passed in gentle currents along the bronchial tubes, sometimes diminishes their contractability. Small doses of strychnia repeated, say from 1-30 to 1-16 of a grain, may co-operate with other remedies.

We think Dr. Watson's method worthy of trial in cases of spasmodic asthma, especially where we have reason to suspect laryngeal disease. We have sometimes seen beneficial effects from large doses of quinine.

